# A Review of the Prevention of Mother-to-Child Transmission of Human T-Cell Lymphotrophic Virus Type 1 (HTLV-1) With a Proposed Management Algorithm

**DOI:** 10.3389/fmed.2022.941647

**Published:** 2022-07-08

**Authors:** Rachael S. Barr, Simon B. Drysdale, Mary Boullier, Hermione Lyall, Lucy Cook, Graham P. Collins, Dominic F. Kelly, Lorna Phelan, Graham P. Taylor

**Affiliations:** ^1^Department of Paediatrics, University Hospitals Bristol NHS Foundation Trust, Bristol, United Kingdom; ^2^Paediatric Infectious Diseases Research Group, Institute of Infection and Immunity, St. George's, University of London, London, United Kingdom; ^3^Oxford Vaccine Group and NIHR Oxford Biomedical Research Centre, Department of Paediatrics, University of Oxford, Oxford, United Kingdom; ^4^Department of Paediatric Infectious Diseases, Imperial College Healthcare NHS Trust, London, United Kingdom; ^5^National Centre for Human Retrovirology, Imperial College Healthcare NHS Trust, London, United Kingdom; ^6^Section of Virology, Department of Infectious Disease, Imperial College London, London, United Kingdom; ^7^Department of Haematology, Oxford University Hospitals NHS Foundation Trust, Oxford, United Kingdom; ^8^Level 2, Children's Hospital, Oxford University Hospitals NHS Foundation Trust, Oxford, United Kingdom; ^9^Department of Obstetrics and Gynaecology, Imperial College Healthcare NHS Trust, London, United Kingdom

**Keywords:** HTLV-1, pregnancy, neonate, antiretrovirals, adult T cell lymphoma/leukemia, prevention of mother-to-child transmission

## Abstract

Human T cell lymphotropic virus type 1 (HTLV-1) is a human retrovirus that is endemic in a number of regions across the world. There are an estimated 5–10 million people infected worldwide. Japan is currently the only country with a national antenatal screening programme in place. HTLV-1 is primarily transmitted sexually in adulthood, however it can be transmitted from mother-to-child perinatally. This can occur transplacentally, during the birth process or via breastmilk. If HTLV-1 is transmitted perinatally then the lifetime risk of adult T cell leukemia/lymphoma rises from 5 to 20%, therefore prevention of mother-to-child transmission of HTLV-1 is a public health priority. There are reliable immunological and molecular tests available for HTLV-1 diagnosis during pregnancy and screening should be considered on a country by country basis. Further research on best management is needed particularly for pregnancies in women with high HTLV-1 viral load. A first step would be to establish an international registry of cases and to monitor outcomes for neonates and mothers. We have summarized key risk factors for mother-to-child transmission of HTLV-1 and subsequently propose a pragmatic guideline for management of mothers and infants in pregnancy and the perinatal period to reduce the risk of transmission. This is clinically relevant in order to reduce mother-to-child transmission of HTLV-1 and it's complications.

## Introduction

Human T cell lymphotropic virus type 1 (HTLV-1) is a human retrovirus that was first identified in 1979 (1). HTLV-1 is widespread throughout the globe, being endemic in regions of Japan, West and Southern Africa, the Caribbean islands, Iran, some parts of South America, Central Australia and Melanesia (2, 3). It is also found in Europe, North America, India and China, with an estimated 5–10 million people infected worldwide (2). Japan has an estimated 800,000 carriers and is the only country with a national antenatal screening programme (4), although French Guiana also undertakes antenatal screening (5). Some countries, such as Brazil, are considering introduction of national screening (6). The recent technical report from the World Health Organization on the global health burden of HTLV-1 infection draws attention to the importance of incorporating HTLV-1 testing into antenatal care. Highlighting cessation of breast-feeding in a public health approach to the elimination of HTLV-1 mother-to-child transmission, whilst also drawing attention to the need for further research in mother to child transmission (3).

It has been estimated that there are 20,000-30,000 HTLV-1 seropositive people living in England and Wales (7). However, without a screening program in the UK, diagnosis of HTLV-1 infections is usually only made in relation to blood and tissue donor screening, related contact tracing, and rare cases of HTLV-1 associated diseases such as HTLV-1 associated myelopathy (HAM) and Adult T-cell leukemia/lymphoma (ATL) which usually have onset in adult life (7). Whilst rarely encountered in pediatric clinical practice, a recent series of ATL cases diagnosed during pregnancy in the UK have emphasized the importance of measures to prevent perinatal transmission in the setting of HTLV-1 associated diseases where due to higher proviral loads, transmission risks are greater than for asymptomatic individuals (8). Many clinicians including obstetricians, midwives, neonatologists and pediatricians, particularly those working in countries with a lower incidence, do not have clinical experience of this condition. This may result in missed opportunities for prevention of mother-to-child transmission (MTCT) of HTLV-1, and have significant implications for the health of the mother and infant in later life.

## Transmission and Disease

The majority of HTLV-1 infections are acquired via sexual transmission in adult life. Sexual transmission between serodiscordant heterosexual couples has been estimated at rates from 0.9 to 2.5 per 100 person-years (9, 10). Higher viral loads in the seropositive partner may be associated with an increased risk of transmission (11). HTLV-1 transmission can also occur by the same routes as other blood-borne viruses including unscreened donated blood, organ donation and injecting drug use (12). Transmission from mother-to-child may occur in the perinatal period via blood, the placenta, the birth canal or breastmilk. The primary route for vertical transmission is via breast milk (~ 80%) and the risk of perinatal transmission by other routes is low in comparison (13).

**Figure 1:**
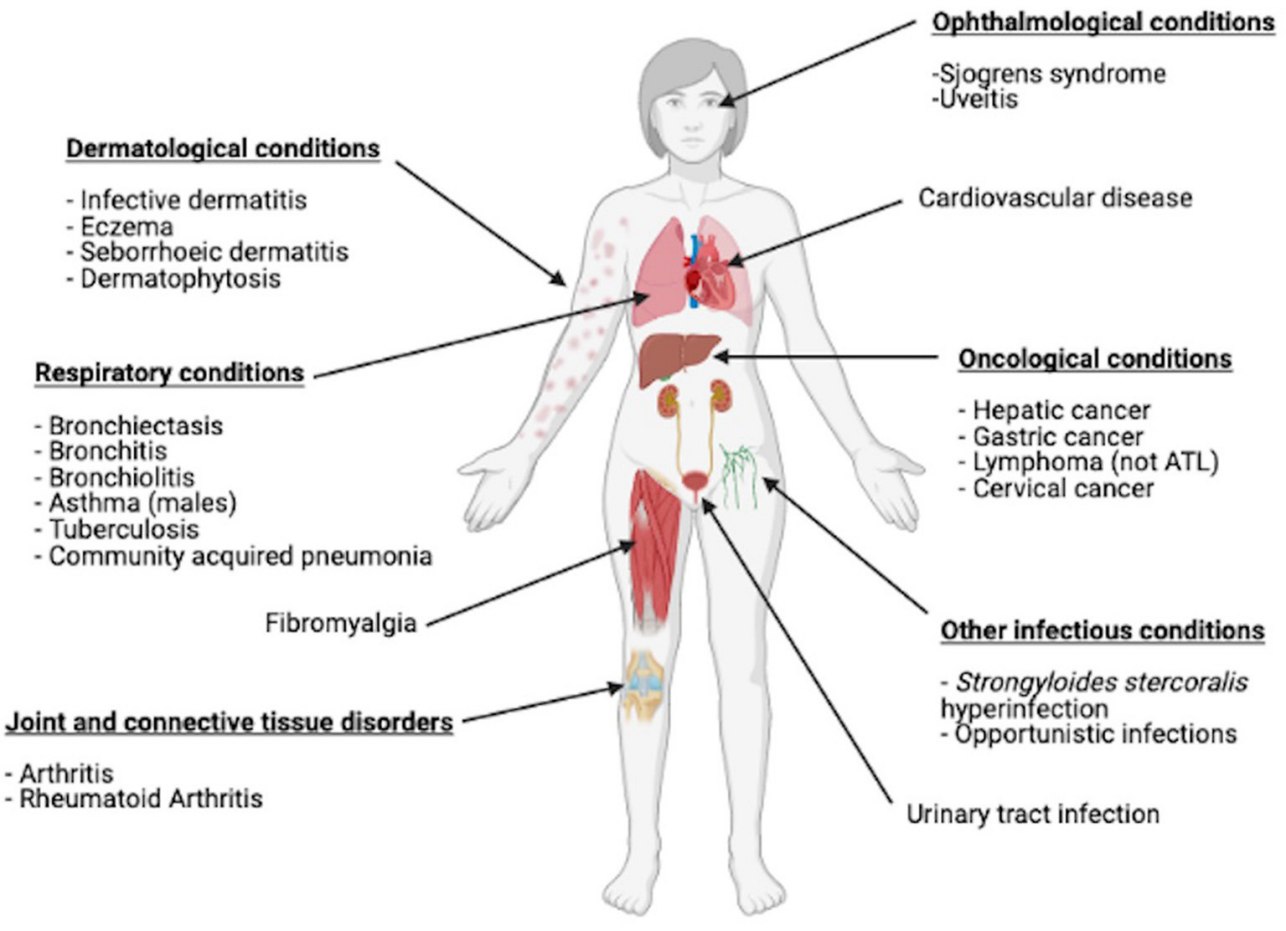
Conditions other than ATL and HAM that have been shown to be linked with HTLV-1 infection in a meta-analysis. Figure created with BioRender.com.

HTLV-1 RNA is rarely detected in plasma (14), which is deemed non-infectious. *Ex vivo* observations revealed that HTLV-1 transmission is through cell-to-cell transfer from infected to uninfected T lymphocytes. HTLV-1 integrates into the host cell DNA, mostly CD4+ T-lymphocytes, where it persists with a single copy per genome/cell (15) and is replicated during cell division. Over 90% of those infected are asymptomatic, however, ~10% develop the high morbidity, high mortality HTLV-1-associated diseases, mostly in adult life. There are two major disease associations, HAM and ATL. ATL is an aggressive CD4+ T-cell malignancy that follows decades of asymptomatic HTLV-1 infection and represents the malignant transformation of an infected CD4+ T cell clone (16). The median survival from diagnosis for the aggressive subtypes (acute and lymphoma) is 8 to 10 months despite intensive therapy (17). Despite novel chemotherapy based approaches, survival has remained unchanged in the ~40 years since HTLV-1 was first described. The overall lifetime risk of HTLV-1 carriers developing ATL is 5% (18). However, acquisition of HTLV-1 as an infant in the perinatal period increases the lifetime risk of developing ATL to 20%. (19) HAM is a progressive condition affecting the spinal cord and causing upper motor neurone symptoms predominantly in the lower limbs. The lifetime risk of developing this complication is estimated at 0.25–3.8% (20). Epidemiological studies have not identified a link between perinatal infection and an increased rate of development of HAM, but rare pediatric cases are reported (21). Looking beyond these known disease associations, a recent meta-analysis showed links between HTLV-1 and increased risk of a further 17 different diseases including dermatological, respiratory, rheumatological and oncological conditions (Figure 1) (22). In this meta- analysis HTLV-1 infection was associated with a 1.57 adjusted Hazard Ratio for mortality unexplained by the risk of ATL. Carriers of HTLV-1 infection are also suspected to be at risk of other opportunistic infections (23) and cardiovascular disease (24) (Figure 1).

Given the link between perinatal transmission and increased lifetime risk of development of ATL, prevention of mother-to-child transmission is a public health priority. Despite this, there is currently no international consensus on the management of pregnant women with HTLV-1.

### Diagnostic Tests and Screening

Timely and accurate diagnosis of HTLV-1 in women of child-bearing age or those who are pregnant is key in the prevention of mother-to-child transmission. Serological testing for detection of antibodies against HTLV-1 is the first line of testing. Options for serological testing include ELISA, particle agglutination, Western blots and chemiluminescence assays (25, 26). Of note, ELISA and particle agglutination tests are seen as screening tests with western blot, chemiluminescence assays and molecular testing being confirmatory.

Rosadas et al. found that both immunological and molecular testing available for HTLV-1 remains reliable during pregnancy (27).

In the UK, HTLV-1 pro-viral load (PVL) testing is undertaken by contacting the National Center for Human Retrovirology (https://www.imperial.ac.uk/medicine/molecular-diagnostic-unit/diagnostic-services/htlv-load-testing-and-genot-yping/). In the proposed management algorithm we suggest antibody testing is performed in all infants of HTLV-1 positive mothers at 18 months of age. This is because by 18 months maternal antibody will have waned and the results are likely to be representative of the child's own antibody response (28). Japan currently recommend this testing is done at 3 years of age however there is a large loss to follow up at 3 years (29).

## Mother-to-Child Transmission Risk Factors and Prevention

A number of factors influence the risk of transmission of HTLV-1 perinatally and each provides an opportunity to decrease the risk to the infant if considered and managed appropriately. A high maternal HTLV-1 PVL (defined as HTLV-1 DNA copies/100 peripheral blood mononuclear cells [PBMCs] expressed as a percentage of cells infected given there is a single copy per cell) has been found to be an independent risk factor for increased transmission to the infant (30). Hisada et al. (30) showed a PVL of >3.0 log_10_ per 10^5^ cells (equivalent to >1%) was associated with a significant increase in the risk of transmission. Transmission risk went from 6.4% when the PVL was <1 to 36.1% when the PVL was 1% or greater. In addition, the same study (30) showed the risk of transmission was negligible when the mother's HTLV-1 antibody titer was <2.0 log_10_ but increased significantly when ≥2.0 log_10_. Symptomatic patients (e.g. those with HAM / ATL) have been shown to have a greatly increased PVL when compared with asymptomatic carriers (31).

We, therefore, recommend stratifying pregnant women who are not symptomatic due to HTLV-1 infection, into low risk (PVL < 1%) and high risk (PVL ≥ 1%) based on their PVL. Women who are symptomatic with ATL should always be considered high-risk, but those with other symptomatic conditions (e.g., dermatitis) may be stratified according to their PVL.

Other risk factors for mother-to-child transmission of HTLV-1 infection include women who have had a previous child with HTLV-1 infection (32), women who are infected with *Strongyloides stercoralis* (33) and genetic risk factors such as an increased risk of transmission with increased concordance in HLA class 1 alleles between mother and child (28, 33, 34). We suggest screening for *Strongyloides stercoralis* in pregnant women newly diagnosed with HTLV-1 infection in pregnancy, or those who are known to have HTLV-1 infection who have not been previously screened for *Strongyloides stercoralis* as it is a treatable risk factor.

Breast milk transmission has been reported to occur in as many as 77% of exclusively breastfed infants compared with 3.3% of non-breastfed infants (35), although other studies have reported lower transmission rates (6). Data from Japan established that there is a direct correlation between duration of breastfeeding and risk of transmission, with longer durations associated with increased transmission (6). The risk of transmission in an infant breastfed for less than 3 months is <2.5% (29, 36). Some studies suggest the risk of MTCT with short term (≤3 month) breastfeeding is not significantly higher than that of infants exclusively formula fed (29, 37). Infants breastfed for less than or equal to 6 months had a transmission risk of 3.9% compared with 20.3% for those breastfed for longer than 6 months (36). Breastfeeding should therefore be avoided if feasible (according to AFASS (Acceptable, Feasible, Affordable, Sustainable and Safe) criteria) (38), or limited to as short a time as possible (e.g., for less than 3 months). However, this should only be applied to populations where the risk of transmitting HTLV-1 infection outweighs the risk of stopping breastfeeding. This decision must be balanced against the known benefits to both infant and mother of breastfeeding and the disadvantages (e.g., cost and need for a clean water supply) associated with formula milk. If a mother decides to breastfeed, or is unable to formula feed safely, then measurement of proviral load in breastmilk should be considered if this is available. In this circumstance, we recommend undertaking HTLV-1 PCR on breastmilk at 1 week and 3 months and every 3 months thereafter as long as breastfeeding continues, done via real-time PCR (39). If the breastmilk HTLV-1 PVL is ≥1%, or if the mother breastfeeds for more than 3 months, there should be further encouragement for breastfeeding to cease if possible, and if it continues then the infant should be considered high risk for developing HTLV-1 infection (6). There is some evidence that freeze-thawing of breast milk provides some benefit in reducing mother-to-child transmission of HTLV-1. Freezing has been shown to affect HTLV-1 infected cells *in vitro* (40) and small studies have shown a reduction in mother-to-child transmission (41). However, this method is not always possible for mothers to employ as it involves freezing milk at −20°C for at least 12 h (40). There are also limited data on pasteurization of breastmilk to reduce HTLV-1 transmission (42).

The route of transmission in infants who are not breastfed is not completely clear, however it is likely to be at the time of delivery. Transplacental infection may occur, but even when infected lymphocytes are detected in cord blood, not all infants become infected (43, 44).

Healthcare professionals should, therefore, consider options to reduce direct transfer of body fluids, particularly blood, at the time of delivery. Currently there are few studies investigating the most effective method of delivery of infants of women who are HTLV-1 carriers to reduce transmission, although (pre-labor) cesarean section may reduce the risk (28).

As in management of HIV-1, antiretroviral medications could be used to reduce HTLV-1 transmission rates in high-risk infants. However, there are few data regarding the use of antiretrovirals for peri-exposure prophylaxis for neonates. We have published a case series (8) of four mothers from the United Kingdom, all with a diagnosis of ATL, where the mothers were treated, and the infants received post-exposure prophylaxis (PEP), with antiretroviral medications. One infant became seropositive at 15 months, the other three remained negative at 6 weeks, 3 months and 6 months of follow up respectively. We are not aware of any other cases in the literature of infants receiving PEP to try to prevent HTLV-1 infection. There are sporadic case reports of women who are carriers of HTLV-1 infection receiving antiretroviral medications to try to prevent MTCT (45). Potential side effects from ARV's also need to be considered when using them in both neonatal and maternal populations.

There are currently no clinical trials of anti-retroviral (ARV) treatment for HTLV-1 infection in pregnancy or for prevention of MTCT of HTLV-1 that we are aware of, although limited *in vitro* data suggest their use may be beneficial (28). Several nucleoside analogs have been demonstrated to have anti-HTLV-1 reverse transcriptase activity *in vitro*, including zidovudine (46). Zidovudine (ZDV) has also been shown to prevent transmission in a rabbit model and has activity against ATL. (47) ZDV with interferon-alpha (IFNα) is an established therapy in leukemic ATL (where available) (48) and is often used as an adjunct to chemotherapy in lymphoma subtype ATL (49). Both ZDV / IFNα are known to be relatively safe in pregnancy, and in cases of ATL have the additional advantage of reducing the risk of mother to infant transmission. Raltegravir (and other integrase inhibitors), also have anti-HTLV-1 integrase activity, and may be used as an adjunct to ZDV (50). While studies (51–53) of antiretroviral therapy have repeatedly failed to show activity *in vivo* in established infection due to the dominance of cell proliferation in the maintenance of the proviral burden, a role in prevention of transmission when given peri-exposure is biologically plausible. As transmission of HTLV-1 is mainly due to cell-to-cell transmission rather than via free virions in plasma, and maternal lymphocytes in the newborn circulation have a longer half-life (54), it is important to note that infant PEP for HTLV-1 should be for a longer duration (6 weeks) than for HIV which is most likely and most often acquired from free virus in plasma (55). Of note, future treatments may include use of neutralizing monoclonal antibodies. None have been used in clinical trials to date however there has been some initial work in animal models (56).

### Guidance for Prevention of Transmission

**Figure 2:**
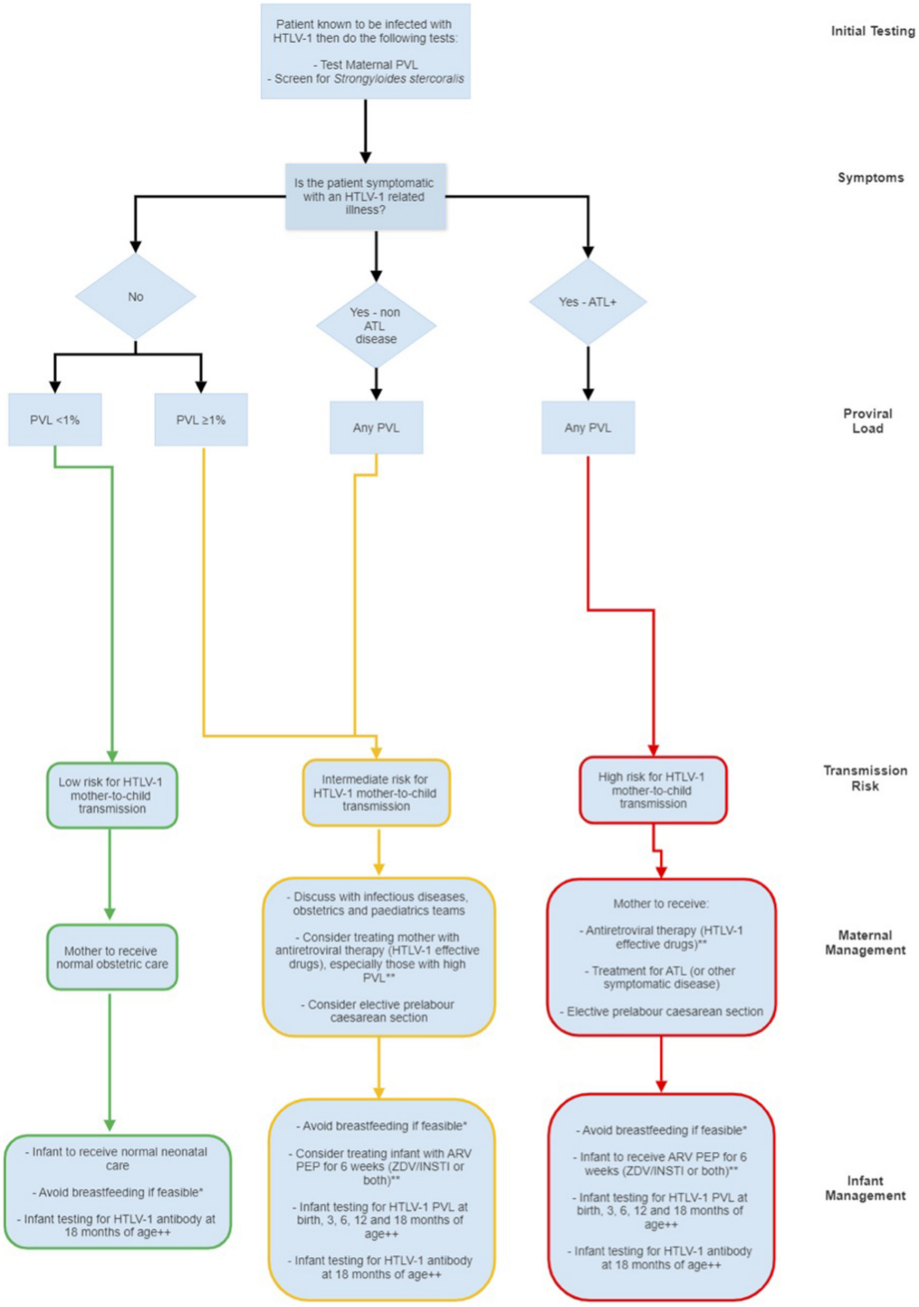
*If a mother decides to breastfeed, consider undertaking breastmilk diagnostics; HTLV-1 PCR at week 1 and 3 months and every 3 months thereafter as long as breastfeeding continues. If the breastmilk HTLV-1 PVL is ≥1% or if the mother breastfeeds for more than 3 months then the infant should have testing for HTLV-1 as per the “high-risk of transmission” arm of the algorithm. **For antiretroviral therapy use same dosing as for treatment (mother) / prevention (infant) of HIV infection (use local guidelines). ^+^Women with leukaemic ATL are theoretically at ultra-high risk of transmission simply because they have higher absolute white cell counts. ^++^If an infant is shown to be infected at any point, there should be HTLV-1 antibody and PVL by PCR testing at 12–18 months of age and then annual quantitative HTLV-1 PVL testing thereafter. Key: ARV, Antiretroviral; ATL, adult T cell Leukemia/Lymphoma; ZDV, Zidovudine; INSTI, Integrase strand transfer inhibitor (e.g. raltegravir); PCR, Polymerase chain reaction; BF, Breastfeeding; PEP, Post-exposure prophylaxis.

Drawing together the available evidence on HTLV-1 transmission, and our experience of managing women and their infants, and bearing in mind the potential role of cesarean section, maternal and neonatal antiretroviral therapy, and avoidance of breastfeeding in preventing mother-to-child transmission of HIV, a similar retrovirus, we propose the following perinatal algorithm (Figure 2) for managing pregnant women with HTLV-1 and their infants.

The algorithm is based on assessment of transmission risk according to clinical disease and PVL: women with no symptoms and PVL < 1%, follow the green path with normal pregnancy, birth and postnatal care, avoiding breast feeding if feasible; women with ATL (whatever the PVL), follow the red path with antiretroviral treatment, elective cesarean section, avoidance of breast feeding and PEP for the infant; women with no symptoms / mild symptoms and PVL >1% follow the yellow path and should be considered for antiretroviral treatment, elective cesarean section, avoidance of breast feeding and PEP for the infant. Clinicians are welcome to contact the National Center for Human Retrovirology (http://www.htlv.eu/) for advice in the management of pregnant women with HTLV-1 infection.

## Conclusion

Data on prevention of mother-to-child transmission of HTLV-1 have been available since the 1980s and are robust, all demonstrating the effectiveness of avoidance of breast-feeding or at least limiting to less than 3 months duration. Mother-to-child transmission is associated with a high risk of subsequent HTLV-1 associated disease especially adult T-cell leukemia. Data on reducing the risk of peripartum transmission are limited, therefore, we propose a pragmatic perinatal algorithm to aid management of these cases, highlighting transmission risks, perinatal diagnostics, and antiretroviral and obstetric interventions. However, further study on best management for the prevention of mother-to-child transmission of HTLV-1 is needed, especially for high-risk pregnancies. A first step would be to establish an international registry of cases and to monitor outcomes for neonates and mothers.

## Author Contributions

RB: writing—original draft and writing-review and editing. SD: conceptualisation, writing—original draft, and writing—review and editing. MB, HL, LC, GC, DK, LP, and GT: conceptualisation and writing—review and editing. All authors contributed to the article and approved the submitted version.

## Conflict of Interest

The authors declare that the research was conducted in the absence of any commercial or financial relationships that could be construed as a potential conflict of interest.

## Publisher's Note

All claims expressed in this article are solely those of the authors and do not necessarily represent those of their affiliated organizations, or those of the publisher, the editors and the reviewers. Any product that may be evaluated in this article, or claim that may be made by its manufacturer, is not guaranteed or endorsed by the publisher.
